# Comparison of the effects of in-person and internet-delivered mindfulness-based stress reduction on the burden of psychosomatic symptoms in nurses

**DOI:** 10.3389/fpsyg.2024.1402075

**Published:** 2024-12-11

**Authors:** Muhmmad Qabil Jamil Al-Badiri, Fataneh Ghadirian, Hosein Zahednezhad, Mahsa Boozari, Mahsa-Sadat Hayati

**Affiliations:** ^1^Department of Management & Psychiatric Nursing, School of Nursing and Midwifery, Shahid Beheshti University of Medical Sciences, Tehran, Iran; ^2^Department of Medical & Surgical Nursing, School of Nursing and Midwifery, Shahid Beheshti University of Medical Sciences, Tehran, Iran; ^3^Department of Psychiatric Nursing, School of Nursing and Midwifery, Iran University of Medical Sciences, Tehran, Iran

**Keywords:** mindfulness-based stress reduction, psychosomatic, medically unexplained symptoms, nurses, telehealth (TH)

## Abstract

**Introduction:**

There is some evidence comparing the efficacy of telehealth to in-person mental health care, but there is limited research specifically comparing these modalities in nurses. The study aimed to compare the effects of Mindfulness-based Stress Reduction (MBSR) and Internet-delivered Mindfulness-based Stress Reduction (iMBSR) on burden of psychosomatic symptoms of nurses working at Al-Alhamzeh general hospital, Aldiwaniyeh, Iraq.

**Methods:**

The study was a semi-experiment study with a pre-posttest design on 72 registered nurses. Subjects were randomly allocated in group A, in-person MBSR and group B, internet-delivered MBSR. Intervention in two groups was held at 8 weekly sessions. The data collection instrument included sociodemographic, Patient Questionnaire Health-15 (PHQ-15), and General Health Questionnaire-12 (GHQ-12). Data were analyzed with SPSS version 24 by descriptive and non-parametric inferential tests.

**Results:**

The study found that 50% of the nurses in both groups reported mild somatic symptoms, and 40% reported moderate symptoms, with the majority showing no signs of mental distress. A more significant reduction in GHQ scores compared to PHQ scores was observed from pre-to post-intervention. Notably, the online MBSR group showed a significant decrease in GHQ scores, both between groups (*p* = 0.04) and within the online MBSR group itself (*p* = 0.02), highlighting the greater impact of the intervention in this group.

**Conclusion:**

The study highlights the positive effects of both in-person and online MBSR interventions on reducing depressive symptoms and improving mental health outcomes among nurses. Online MBSR, in particular, shows promise in addressing medically unexplained symptoms and enhancing mental well-being.

## 1 Introduction

Nurses are at a high risk of experiencing stress due to the demanding nature of their work. Stress, in this context, refers to a physical, emotional, or psychological response to perceived challenges or threats, which can result in a variety of stress reactions. These reactions include anxiety, irritability, and difficulty concentrating, as well as more severe consequences like burnout or physical health problems (Okuhara et al., 2021). Stress can stem from cognitive appraisals of threats or excessive demands, and it is particularly common among healthcare professionals who often operate in high-pressure environments with inadequate resources.

In the nursing profession, the relationship between stress and physical symptoms is particularly significant. Physical symptoms that arise from mental or emotional strain—rather than from a purely physical cause—are categorized as psychosomatic. Common stressors for nurses include insufficient staffing, heavy workloads, poor teamwork, lack of proper training, limited supervision, and workplace conflicts. These factors can lead to a range of psychosomatic symptoms, such as sleep disturbances, chronic fatigue, headaches, musculoskeletal pain, and gastrointestinal discomfort (Gu et al., 2019). The intensity of these symptoms is closely tied to the perceived severity of the stressor. Estimates suggest that between 14.68% and nearly 50% of nurses may experience psychosomatic symptoms related to occupational stress (Busch et al., 2022; Akodu and Ashalejo, 2019).

Addressing this issue is crucial for healthcare organizations. A range of psychological interventions can help manage stress and alleviate psychosomatic symptoms in nurses, including mindfulness-based techniques, relaxation strategies, cognitive-behavioral therapy (CBT), stress management training, support groups, and Employee Assistance Programs (EAPs) (Fava et al., 2017). Among these, Mindfulness-Based Therapy (MBT) has garnered significant attention. MBT incorporates mindfulness meditation and CBT techniques, helping individuals focus on present-moment awareness without judgment. This approach has been shown to reduce stress, anxiety, and depression, while also decreasing physical symptoms like pain and fatigue (Ivtzan and Lomas, 2016).

Evidence regarding the effectiveness of MBT has been mixed but generally positive. A meta-analysis found that MBT outperformed psychological education (Hedges’ g 0.61), supportive therapy (Hedges’ g 0.37), relaxation techniques (Hedges’ g 0.19), and imagery or suppression strategies (Hedges’ g 0.26) (Khoury et al., 2013). However, the effectiveness of MBT appears to vary depending on the type of symptoms being targeted. While psychological symptoms such as anxiety and depression show significant improvement, the impact on psychosomatic symptoms has been less conclusive, with moderate effect sizes in comparison to treatments for purely psychological disorders (Khoury et al., 2013).

Mindfulness-Based Stress Reduction (MBSR), a specific form of MBT, has demonstrated potential benefits for reducing psychosomatic symptoms, including medically unexplained symptoms and somatic symptom disorder (Zargar et al., 2021; Aktaş et al., 2019). MBSR has been shown to enhance the quality of life, alleviate stress, and improve symptoms related to anxiety and depression (Wexler and Schellinger, 2023). While there is considerable evidence supporting MBSR for psychological symptoms, the research on its effectiveness for physical and psychosomatic symptoms, particularly in nursing contexts, is still developing.

In recent years, telehealth has emerged as a viable platform for delivering psychological interventions, offering flexibility and increased accessibility. Telehealth can eliminate travel barriers for nurses and provide cost-effective alternatives to in-person therapy. While studies suggest that telehealth can be as effective as traditional face-to-face therapy for managing anxiety and depression (Marton and Kanas, 2016; Bulkes et al., 2022), there is a lack of research focusing specifically on psychosomatic symptoms among nurses and comparing the outcomes of in-person and online interventions in this group.

The healthcare system in Iraq presents unique challenges due to economic and social factors, including resource limitations and the lingering effects of conflict. There is a significant gap in the literature regarding the prevalence of psychosomatic symptoms among Iraqi nurses and the effectiveness of tailored interventions in this context. This study aims to address this gap by comparing the efficacy of Mindfulness-Based Stress Reduction (MBSR) delivered in traditional and internet-based formats (iMBSR) among nurses in Iraqi hospitals. By investigating these interventions, we seek to provide evidence-based strategies that are both effective and accessible, with the potential to improve the well-being of nurses operating under high levels of occupational stress.

The study is guided by the following research questions:

1. Research Question 1: How effective is MBSR in reducing psychosomatic symptoms among nurses compared to a control group?
2. Research Question 2: Is iMBSR as effective as traditional MBSR in alleviating psychosomatic symptoms among nurses?
3. Research Question 3: What are the differences in the mechanisms of action between traditional MBSR and iMBSR in managing stress-related symptoms?

Based on these questions, the study hypothesizes that:

1. Hypothesis 1: Nurses who participate in the MBSR intervention will experience a greater reduction in psychosomatic symptoms compared to those in the control group.
2. Hypothesis 2: There will be no significant difference in the reduction of psychosomatic symptoms between nurses receiving MBSR and those receiving iMBSR.
3. Hypothesis 3: The mechanisms by which MBSR and iMBSR reduce psychosomatic symptoms will differ, with iMBSR potentially offering greater accessibility and flexibility, leading to enhanced adherence and convenience for nurses.

## 2 Materials and method

### 2.1 Design

The study was a semi-experiment study with a pre-post-1 test design, experimental group A and experimental group B.

### 2.2 Participants

The study population was all nurses who were working at Al-Hamzeh general hospital at Diwaniyeh, Iraq. The intervention was conducted from December 2023 to March 2024. Al-Hamzeh General Hospital is situated about 25 km south of Al Diwaniyah and 175 km south of Baghdad, on the Diwaniya Channel branch of the Euphrates. The hospital provides emergency care, intensive care unit (ICU) services, and operating theaters.

Subjects were part of the study population (nurses) that meet our inclusion criteria: Nurses with BSc license; Aged between 18–65; Work experience more than 1 year (It takes maximum of 1 year for a person to adapt psychologically with a new environment); No having known psychiatric disorders such as Schizophrenia, MDD, bipolar disorder, Obsessive compulsive disorder (OCD) and etc. based on self-report; No having co-morbid serious medical diseases (such as multiple sclerosis, cancer and so on); No having a somatic or psychiatric disorder explaining their somatic symptoms; No having PHQ-15 < 5. The exclusion criteria were: Participants who fill the questionnaires incompletely, will be excluded (More than 10% of questions); Happening a severe stressor during the study’ intervention period; Not attending or being absent in more than 2 sessions of interventions; Not having active participation in completing assignments (Based on written reports); Refuse to continue the study.

Based on Wortman et al. (2019) study effect Sizes (ES) of psychosomatic therapy are *d* = 0.79 for perceived symptom severity and *d* = 0.54 and *d* = 0.56 for, respectively, somatization and health change. The sample size was calculated using G-power 3.1. Based on an estimated moderate effect size of 0.54, alpha level = 0.05, power = 0.95 in an independent t-test analysis, we estimated that a sample size of 17 participants per group need be recruited. With a drop rate of 20% and reaching to parametric analysis, the final sample size in each group was 36 (*N* = 72).

**Figure 1:**
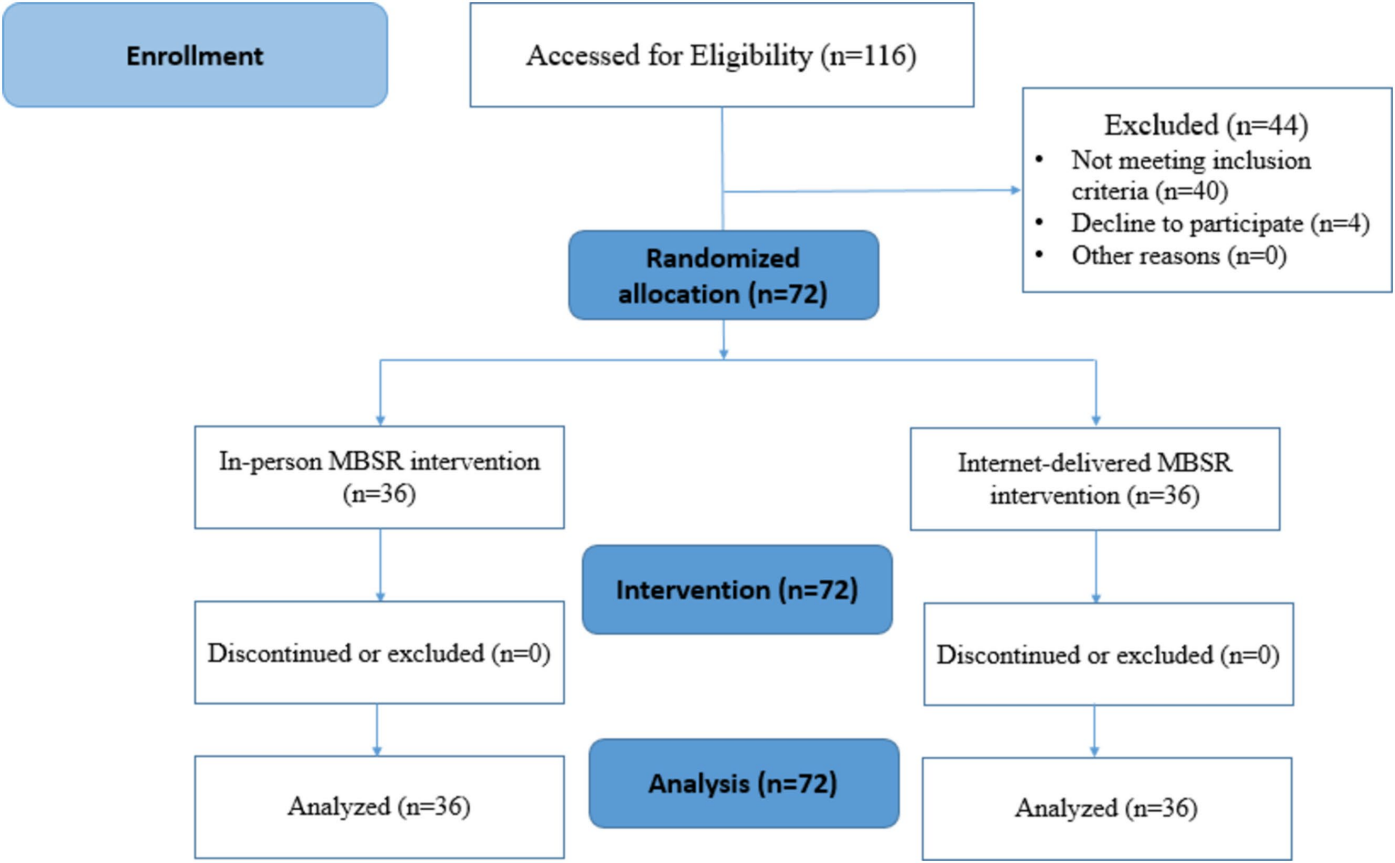
CONSORT flow diagram of sample selection.

Recruiting participants for the study involved three stages: identifying eligible participants, approaching them, and obtaining their consent to join the study. A probability sampling method was used, with a simple random selection of nurses from different hospital wards. Once selected, the nurses were randomly assigned to one of two groups (A or B). The randomization was conducted using a computer software program, Research Randomizer, which generated a random sequence to assign participants. This software, a free online tool, ensured unbiased allocation by generating random numbers and assigning participants to experimental groups (see Figure 1). Additionally, participants were allocated sequentially according to the random sequence generated by the program, ensuring that the process was fully randomized and transparent.

### 2.3 Measures

The study’ instrument had 3 sections: Socio-demographic section, Patient Questionnaire Health-15 (PHQ-15), and General Health Questionnaire-12 (GHQ-12). Permission has been obtained from the authors of the GHQ-12 and PHQ-15 scales for their use in this study.

#### 2.3.1 Socio-demographic questionnaire

The sociodemographic questionnaire of the study was composed of the following items: age, sex (male, female, transgender or other gender identity), the ward of working, history of working (in years), marriage status, educational level (BSc, MSc, PhD), work hours per week, interest in nursing as a profession, satisfaction level with the work atmosphere.

#### 2.3.2 Arabian-version of Patient Questionnaire Health-15 (PHQ-15)

The Patient Health Questionnaire-15 (PHQ-15) is a brief, self-administered questionnaire that evaluates the severity of somatic symptoms. It comprises 15 somatic symptoms, each scored from 0 (“not bothered at all”) to 2 (“bothered a lot”). It is intended to function as a continuous measure of somatic symptom severity and can be used to screen for somatization concerns and monitor symptom severity. The total PHQ-15 score ranges from 0 to 30, with higher scores indicating a greater severity of somatic symptoms. The PHQ-15 score is divided into several categories to illustrate more clearly the severity of somatic symptoms. Scores of ≥5, ≥10, and ≥ 15 represent mild, moderate, and severe levels of somatization, respectively. Each item on the PHQ-15 is rated on a 3-point scale (0 = not bothered at all; 1 = bothered a little; 2 = bothered a lot) (Kocalevent et al., 2013). The Arabic version of PHQ-15 was translated exactly like the English-version (with 15 items). The PHQ-15 has been found to be a reliable and valid self-report measure for somatization syndromes in the general population. It has good internal consistency (*α* = 0.80) when used by adults, and good test–retest reliability and convergent validity with other measures of somatic symptom severity (Kocalevent et al., 2013; Kroenke et al., 2002). Overall, the PHQ-15 is considered a valid and moderately reliable questionnaire for evaluating the severity of somatic symptoms. The validity and reliability of the Arabic version of the PHQ-15 have been evaluated in a Saudi sample. The study found that the Arabic version of the PHQ-15 is a valid and reliable tool to screen for depression, anxiety, somatic, panic, eating, and alcohol abuse disorders (AlHadi et al., 2017). The reliability of the questionnaire in the study was determined by Cronbach coefficient as 0.89.

#### 2.3.3 The Arabic version of General Health Questionnaire-12 (GHQ-12)

The General Health Questionnaire-12 (GHQ-12) is a self-administered screening tool designed to detect current state mental disturbances and disorders. It was developed by David Goldberg, a British psychologist, in the 1970s. The GHQ-12 consists of 12 statements to which respondents indicate agreement on a four-point scale (0 = Not at all; 3 = More than usual). The GHQ-12 is thought to be helpful in some cultures as it does not have as many somatic items and, theoretically, may be better at detecting mental problems among populations with lots of physical comorbidities. The total GHQ-12 score ranges from 0 to 36, with higher scores indicating more severe symptoms of psychological distress. The GHQ-12 score can be interpreted as follows: Scores of 0–11 are considered typical, Scores of 12–15 suggest evidence of distress, Scores of 16–20 indicate moderate distress, Scores of 21 or higher indicate severe distress (26). The Arabic version of PHQ-15 was translated exactly like the English-version (with 12 items). The General Health Questionnaire-12 (GHQ-12) is a widely used tool for measuring the mental health status of respondents. GHQ-12 has been found to be a reliable and valid tool for measuring psychological distress in various populations (Hankins, 2008; Gnambs and Staufenbiel, 2018; Campbell et al., 2003). A study conducted on a sample of university students in the United Arab Emirates found that the Arabic version of GHQ-12 is reliable, with a Cronbach’s alpha of 0.86. The study also found that the best balance between sensitivity and specificity was found at the GHQ-12 cut-off point of 15/16 (Daradkeh et al., 2001). The reliability of the questionnaire in the study was determined by Cronbach coefficient as 0.91.

### 2.4 Intervention

The study protocol was initially reviewed and approved by the Institutional Review Board (IRB) and the Ethics Committee of the School of Nursing and Midwifery at Shahid Beheshti University of Medical Sciences (Ethical code No. IR.SBMU.PHARMACY.REC.1402.183). Once formal approvals were secured, the researcher contacted eligible nurses from Al-Hamzeh Hospital via email or WhatsApp. From the eligible nurses, 72 were randomly selected and allocated to one of two groups (A or B) using the Research Randomizer, an online tool for simple random assignment. Participants were then asked to complete baseline questionnaires, including the Socio-demographic questionnaire, GHQ-12, and PHQ-15 (T0).

For group A, in-person MBSR therapy sessions were conducted over 8 weekly sessions, each lasting 1.5 to 2 h. In contrast, group B participated in iMBSR sessions delivered online via Google Meet or Skype. The iMBSR sessions were structured similarly to the in-person sessions, with each session covering key MBSR topics, including living optimistically, finding meaning in life, flexibility, the power of mindfulness, and achieving personal transformation through daily improvements. Both groups received identical content (see Table 1 for session topics). Each session included guided mindfulness exercises, reflective discussions, and educational materials, ensuring participants in the online group had an experience equivalent to that of the in-person group.

**Table 1:** The content of sessions of MBSR.

No.	Head topic	Contents
1	Orientation and Introduction to Mindfulness	Explanation of the MBSR program and its benefits Introduction to mindfulness and its application in daily life Body scan practice
2	Perceiving the Breath and the Body	Mindful breathing exercises Body scan practice Gentle yoga or stretching
3	Mindful Movement and Body Awareness	Mindful walking or standing meditation Gentle yoga or stretching Body scan practice
4	Working with Thoughts and Emotions	Noting thoughts and emotions without judgment Loving-kindness meditation Body scan practice
5	Stress and Communication	Mindful communication exercises Exploring stress triggers and responses Body scan practice
6	Cultivating Resilience and Compassion	Loving-kindness meditation Self-compassion practice Body scan practice
7	Integrating Mindfulness into Daily Life	Mindful eating Mindful walking or standing meditation Body scan practice
8	Review and Integration	Reflection on the MBSR journey Creating a personal mindfulness plan Body scan practice

Participants in both groups were assigned weekly tasks, which they submitted to the researcher via WhatsApp. The research team assessed these assignments and provided individualized feedback. After completing the 8-week intervention, participants from both groups were asked to fill out the GHQ-12 and PHQ-15 again, immediately following the intervention (T1). This consistent structure between the in-person and online formats ensured that both groups received the same content and assignments, regardless of the delivery mode.

### 2.5 Data analyses

Data collected with the study instruments were coded in the IBM^®^ SPSS^®^ software platform version 24. The types of the main variables of the study were: Somatic symptom severity (discrete quantitative and ranked qualitative) and psychological distress severity (discrete quantitative and ranked qualitative).

Descriptive statistics provided a summary of data in the form of mean, median, mode, Variance, and standard deviation (SD). With inferential statistics, data were analyzed from a sample to make inferences in the larger collection of the population. The purpose was to answer or test the hypotheses regarding the impact of in-person MBSR versus internet-delivered MBSR on psychosomatic symptoms among nurses. The Shapiro–Wilk normality test revealed a non-parametric distribution for all results (*p* > 0.05). The Fisher’s exact test, Mann–Whitney U, Friedman and Wilcoxon tests were employed to assess the mean differences in GHQ and PHQ and socio-demographic results within and between the two groups.

## 3 Results

### 3.1 Socio-demographic characteristics

A total of 72 subjects participated in the study, with 36 subjects in the in-person intervention group (group A) (50%) and 36 subjects in the internet-delivered intervention group (group B) (50%). The mean age of all participants was 34.00 ± 7.62, ranging from 20 to 51 years old. The majority were female (*n* = 48, 66.7%) and most held a BSc degree, with only 2 individuals at the PhD level in group A. Approximately half of the participants in both groups had an intermediate level of interest in the nursing profession, while around 40% had a high level of interest. More than half of the participants reported an intermediate level of satisfaction with their work environment in both groups. Statistical tests, including Mann–Whitney U, Fisher’s exact test, and Kruskal-Wallis test, did not reveal any significant differences in socio-demographic characteristics between the two groups (*p* > 0.05) (Table 2).

**Table 2:** Demographics characteristics compared between the two groups.

Variables	Group A	Group B	Statistics
	In-person MBSR	Online MBSR	
	Mean ± SD	Mean ± SD	Value, *p*
Age	36.00 ± 8.31	32.00 ± 6.37	−1.78*, 0.07
Working years	11.08 ± 8.19	10.03 ± 6.14	−0.26*, 0.79

		*n* (%)	*n* (%)	Value, df, *p*
Sex	Male	9 (25.0)	15 (41.7)	2.25**, 1, 0.21
	Female	27 (75.0)	21 (58.3)	
The working ward	Childbirth	4 (11.1)	4 (11.1)	1.38***, 1, 0.23
	Surgery	9 (25.0)	5 (13.9)	
	Heart	4 (11.1)	5 (13.9)	
	Infectious diseases	7 (19.4)	6 (16.7)	
	Major operations	5 (13.9)	3 (8.3)	
	Emergency	7 (19.4)	13 (36.1)	
Interest to nursing profession	At all	4 (11.1)	3 (8.3)	0.46***, 1, 0.49
	Moderate	15 (41.7)	20 (55.6)	
	High	17 (47.2)	13 (36.1)	
Satisfaction degree	Very dissatisfied	8 (22.2)	2 (5.6)	3.22***, 1, 0.07
	Dissatisfied	3 (8.3)	3 (8.3)	
	Neutral	22 (61.1)	26 (72.2)	
	Satisfied	2 (5.6)	3 (8.3)	
	Very satisfied	1 (2.8)	2 (5.6)	

*Mann–Whitney U value (Z).

**Fisher’s exact value (X^2^).

***Kruskal-Wallis value (H).

### 3.2 Comparison of score changes of psychosomatic symptoms between the two groups

**Figure 2:**
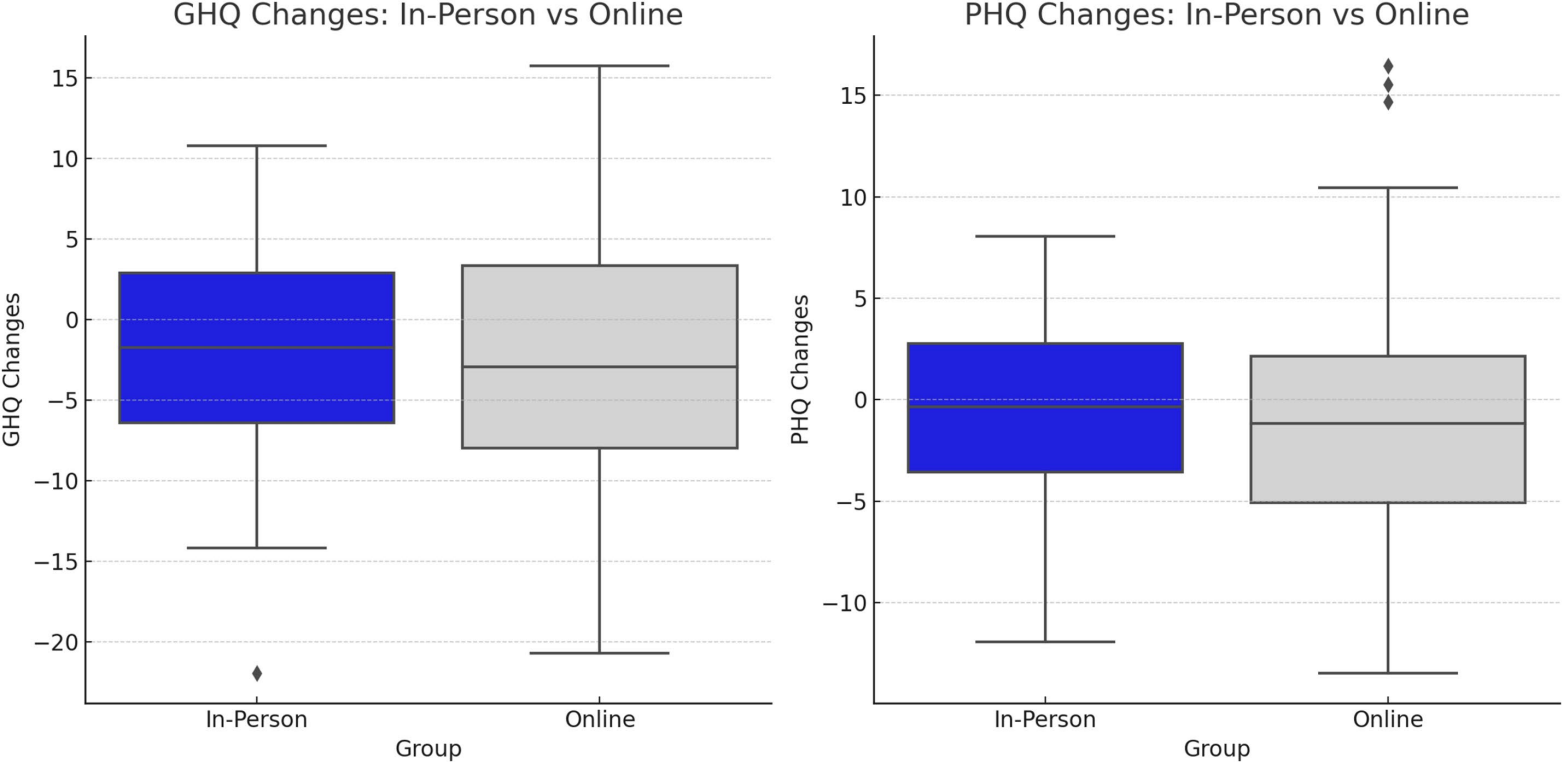
GHQ and PHQ score changes between the two groups, before and after the intervention.

The baseline scores of PHQ and GHQ were compared between groups to check for initial homogeneity. No significant baseline differences were found (PHQ pre-intervention: mean = 8.89, SD = 2.12 for group A; mean = 9.19, SD = 3.07 for group B; *p* = 0.85. GHQ pre-intervention: mean = 10.03, SD = 5.28 for group A; mean = 10.03, SD = 4.96 for group B; *p* = 0.82). Post-intervention, the findings revealed no significant differences between the two intervention groups (A and B) in somatic symptoms (*p* > 0.05). The results indicated a non-significant decrease in PHQ mean scores in the online MBSR group from 9.19 ± 3.07 to 7.58 ± 3.61 (*p* = 0.17). The decline in GHQ scores in both groups from pre-to post-intervention was more pronounced than the PHQ scores, with a significant decrease in the online MBSR group (between groups *p* = 0.04, within-group *p* = 0.02; effect size Cohen’s d = 0.10) (Figure 2, Table 3).

**Table 3:** Comparison of PHQ and GHQ scores between the two groups, before and after the intervention.

Variable		Group A	Group B	Z*	*p*
		In-person MBSR	Online MBSR		
		Mean ± SD	Mean ± SD		
PHQ	Before	8.89 ± 2.12	9.19 ± 3.07	−0.18	0.85
	After	8.06 ± 3.66	7.58 ± 3.61	−0.71	0.47
	Changes	−0.83 ± 4.19	−1.61 ± 5.39	−0.91	0.36
Comparison of before and after within groups (*z*, *p*)		−1.34, 0.17	−1.36, 0.17		
GHQ	Before	10.03 ± 5.28	10.03 ± 4.96	−0.22	0.82
	After	8.58 ± 4.22	7.17 ± 5.00	−2.01	0.04**
	Changes	−1.44 ± 6.52	−2.96 ± 7.23	−0.70	0.48
Comparison of before and after within groups (*z*, *p*)		−1.71, 0.08	−2.18, 0.02**		

*Mann–Whitney U value (Z).

**Significant at *p* < 0.05 level.

### 3.3 Comparison of level changes of psychosomatic symptoms between the two groups

Half of the nurses in both groups experienced mild somatic symptoms, with 40% reporting moderate symptoms, while the majority showed no signs of mental distress. The Mindfulness-Based Stress Reduction (MBSR) intervention demonstrated improvements in reducing moderate somatic symptoms among nurses in both groups. Post-intervention, a statistically significant improvement in PHQ levels was observed for the online MBSR group (mean decrease: −4.00, SD = 11.10; *p* = 0.04), whereas the in-person MBSR group did not show a significant change (*p* = 0.05). Regarding GHQ levels, the data suggested a general decrease in mild mental distress, but no significant differences were detected between or within groups (see Table 4).

**Table 4:** Comparison of PHQ and GHQ level between the two groups, before and after the intervention.

Variable		Group A		Group B		Comparison between (H, df, *p*)	
		In-person MBSR		Online MBSR			
		(*n*,%)		(*n*,%)			
		Before	After	Before	After	Before	After
PHQ	No problem	0 (0.0)	6 (16.7)	0 (0.0)	5 (13.9)	0.01, 1, 089	0.001, 1, 0.97
	Mild	20 (55.6)	20 (55.6)	21 (58.3)	21 (58.3)		
	Moderate	16 (44.4)	8 (22.2)	14 (38.9)	10 (27.8)		
	Severe	0 (0.0)	2 (5.6)	1 (2.8)	0 (0.0)		
Comparison of before and after, within groups (X^2^*, df, *p*)		3.87, 1, 0.05		4.16, 1, 0.04**			
GHQ	Typical	21 (58.3)	28 (77.8)	23 (63.9)	28 (77.8)	0.13, 1, 0.71	0.001, 1, 0.98
	Evidence of distress	10 (27.8)	5 (13.9)	8 (22.2)	5 (13.9)		
	Moderate	3 (8.3)	2 (5.6)	2 (5.6)	3 (8.3)		
	Higher severe	2 (5.6)	1 (2.8)	3 (8.3)	0 (0.0)		
Comparison of before and after (X^2^, *p*)		1.80, 1, 0.18		2.00, 1, 0.15			

* Friedman test value (X^2^).

**Significant at *p* < 0.05 level.

### 3.4 Significant relationships of PHQ and GHQ changes

In the online MBSR group, there was no significant relationship between changes in PHQ and GHQ scores (r = −0.01, *p* = 0.94). In contrast, a positive and significant correlation was found in the in-person MBSR group (r = 0.58, *p* = 0.0001), indicating a moderate effect size. No significant associations were observed between changes in PHQ and GHQ scores with socio-demographic variables, except for a significant correlation between GHQ changes and interest in the nursing profession within the online MBSR group (group B).

## 4 Discussion

The present study was conducted with the aim to determine the effect of in-person mindfulness-based Stress Reduction (MBSR) and internet-delivered Mindfulness-based Stress Reduction (iMBSR) on burden of psychosomatic symptoms of nurses working at Al-Alhamzeh general hospital, Aldiwaniyeh, Iraq. The findings suggest that MBSR interventions, both in-person and online, are associated with positive effects on reducing depressive symptoms and improving mental health outcomes. Specifically, the data showed a decreasing trend in somatic symptoms over the course of the 8-week intervention, particularly among nurses with moderate levels of somatic symptoms. This supports Hypothesis 1, indicating that MBSR contributes to a reduction in psychosomatic symptoms. The results also revealed that while both in-person and online MBSR interventions led to improvements, the iMBSR group demonstrated more significant changes in MUS symptoms. This supports Hypothesis 2, suggesting no major difference between MBSR and iMBSR, although the online format showed slightly better outcomes for certain variables, potentially due to greater accessibility and flexibility. The study identified that the mechanisms of symptom reduction differed between traditional MBSR and iMBSR. In the online group, the association between improvements in somatic symptoms and reductions in mental distress was more pronounced. This supports Hypothesis 3, suggesting that iMBSR’s accessibility might enhance adherence and engagement, leading to more noticeable changes in psychosomatic symptoms.

### 4.1 MBSR intervention and medically unexplained somatic (MUS) symptoms in nurses

At first, our study revealed that nearly 90% of nurses experience mild to moderate MUS symptoms (MUSS). The literature suggests that the prevalence of MUSS in nurses varies between 30 and 50% (Vermeir et al., 2021). It seems that there is a lack of knowledge about MUS symptoms in nurses. There is moderate evidence to suggest a high prevalence of work-related musculoskeletal diseases in nurses (Sun et al., 2023). Sun et al. (2023) believed that higher musculoskeletal diseases are associated with lower psychological resilience and job satisfaction in nursing roles. This finding is congruent with our study. Our results showed that only 8.4% of nurses in group A and 13.9% of them in group B were satisfied with their working situation.

The evidence suggests that the meaning of suffering from MUSS is like a struggle and strain in the sense of self (Polakovská and Řiháček, 2022). Zurlo et al. (2020) assumed that work–family conflicts of nurses are significantly related to somatization of male and female nurses (Zurlo et al., 2020). Seo et al. (2023) claimed that shift workers experience fatigue and somatization associated with their sleep disturbances and depression (Seo et al., 2023).

Secondly, the findings showed that despite non-significant results of MBSR on somatic symptoms scores in two groups, MBSR had remarkable decreasing effects on moderate somatic symptoms level in both groups and resulted in significant changes in level of somatic symptoms experience in online delivered MBSR (group B). The results in both groups showed that the MBSR did not affect nurses with mild somatic symptoms.

Although there is scarce evidence in the literature about MBSR and somatic symptoms in nurses, there is a bunch of knowledge affirming the remarkable effects of MBSR on psychological status and stress in nurses (Ramachandran et al., 2023). This finding of our study is incongruent with findings of the systematic review of Billones et al. (2020) on effects of mindfulness-based interventions (MBIs) on MUS symptoms. They declared that MBIs had large effect sizes on MUSS ranging from 0.62 to 0.82 (Billones et al., 2020).

There are two issues that might be considered: the first is that the scores and the level of somatic symptoms in the study during the 8-week sessions have had a decreasing trend. It may suggest that for attaining more remarkable and significant results, it is needed to extend or modify MBSR for nurses. This assumption is also discussed by Billones et al. (2020). They believed that a manualized Mindfulness-Based Intervention (MBI) incorporates the four essential elements is crucial for its effectiveness. These elements consist of psycho-education sessions to better understand medical symptoms, practicing awareness, nonjudgmental observation of experiences in the moment, and self-compassion. The success of various mindfulness interventions requires addressing identified gaps, including home-based practice monitoring, competency training for mindfulness teachers, and reliable psychometric properties to measure mindfulness practice (Billones et al., 2020).

Second, the results of the study indicate that the nurses in the online MBSR group had significant changes in medically unexplained symptoms compared to the in-person MBSR group. This suggests that online MBSR may be an effective intervention for reducing medically unexplained symptoms in nurses. In line with our finding, Merrigan et al. (2023) stated that the virtual 8-weekly mindfulness training to health professionals improved their respiration rates, perceived stress, and resilience (Merrigan et al., 2023).

One possible explanation for these findings is that online MBSR may provide greater accessibility and convenience for nurses who may not be able to attend in-person sessions due to scheduling conflicts or geographical limitations. Additionally, online MBSR may offer a more flexible and personalized approach to learning and practicing mindfulness techniques, which may be more appealing to some individuals. Another possible explanation is that the online format may allow for a more consistent and structured approach to learning and practicing mindfulness techniques, which may be more effective in reducing medically unexplained symptoms. Further research is needed to confirm these findings and to explore the underlying mechanisms that may be contributing to the observed differences between online and in-person MBSR. Nonetheless, the results of this study suggest that online MBSR may be a promising intervention for reducing medically unexplained symptoms in nurses.

### 4.2 MBSR intervention and mental distress symptoms in nurses

The study results indicated a—decrease in GHQ scores post-intervention in both groups, with a significant difference between the two groups. Online MBSR was found to be more effective than in-person MBSR, showing a small effect size (d = 0.11). This aligns with Chen et al.’s (2020) meta-analysis, which highlighted the positive impact of MBSR on reducing mental distress and enhancing mental resilience in nurses (Chen and Cui, 2020). Mindfulness interventions have been associated with decreased nurse distress perceptions (Vaclavik et al., 2018).

On the contrary, some systematic reviews suggest that MBSR may not be as effective in reducing burnout or enhancing resilience among healthcare providers. However, it has shown effectiveness in promoting self-compassion and mindfulness among healthcare professionals. Additionally, evidence indicates that brief MBSR can be as effective as the traditional 8-session format (Kriakous et al., 2021). The reduction in mental distress scores and small effect size in this study suggest that while MBSR is recognized as beneficial for nurses’ mental health, certain factors may complicate the perception of distress reduction.

For example, the results showed that the GHQ scores before the intervention is associated to age (r = −0.29, r = 0.01) and working years (r = −0.29, r = 0.01). This showed that the mental distress experienced by nurses decreases with an increase in age and working years. In addition, the results showed that the GHQ change overall is impacted by interest in nursing profession. In congruent of our study, Ghazawy et al. (2021) stated that more than half of nurses are dedicated to their work, but they need to have the needed resources, supportive environments, and performance feedback in order to balance between work demands and the feeling of fulfillment and reduced turnover rates.

In congruent with our study Taylor et al. (2022) in a study entitled “Health Care Workers’ Need for Headspace” revealed that depression, anxiety and stress of health workers could effectively reduce by a digital mindfulness program. They discussed that in-person mindfulness-based interventions can reduce health care worker stress but are not widely available or accessible to busy health care workers. They suggest that due to lack of accessible, affordable, and effective approaches to reducing stress of health workers and despite that the result of digital mindfulness showed a small effect size, but could have a population-based benefits (Taylor et al., 2022).

### 4.3 MBSR intervention and body–mind complex relationships

The study results indicated a significant association between changes in somatic symptoms and mental distress, particularly in the context of an online group. Interestingly, despite the random allocation of nurses into groups, it was observed that nurses in group A may have exhibited more somatization traits.

#### 4.3.1 Somatization and its psychological implications

Somatization involves the manifestation of psychological and emotional distress through physical symptoms. It can serve as a defense mechanism with various functions. Focusing on bodily sensations may help individuals avoid confronting painful emotions or conflicts, or it could serve to deflect aggressive thoughts by portraying oneself as physically vulnerable.

#### 4.3.2 Somatization and alexithymia

Research has linked somatization to alexithymia, which refers to a limited ability to recognize and express emotions effectively. This connection underscores the role of emotional regulation in somatic symptom presentation (Raffagnato et al., 2020).

#### 4.3.3 Maladaptive emotion regulation and symptom formation

Some perspectives view somatization as a maladaptive method of emotion regulation, contributing significantly to the development and persistence of medically unexplained symptoms (MUS). Understanding somatization as a psychological factor is crucial in addressing symptomatology (Merced, 2022).

#### 4.3.4 Interceptive awareness and somatization

Historically, somatic experiences and bodily signals ae conceptualized negatively. With emerging the clinical utility of mindfulness, the notion of problematic nature of high somatic focus has questioned. Some believe that high interceptive awareness lead to somatization (cognitive behavioral model) and other calms that low interceptive awareness lead to somatization (predictive model). Hohl (2022) believed that both overly low and overly high levels of interceptive awareness were expected to be associated with a higher tendency toward experiencing somatization.

MBSR intervention and potential risks: Our study revealed minor evidence that some individuals who underwent MBSR in-person reported increased somatic symptoms post-intervention. While MBSR is generally considered safe, it is essential to acknowledge the existence of potential risks. These risks include physical discomfort during yoga practice, psychological distress from participation, and challenges in dedicating time and space for practice.

For the final, the results indicate that iMBSR shows promise as an effective and accessible intervention for providing psychological support and self-help training to nurses. To enhance its impact on somatic symptoms and psychological resilience in nurses, adjustments to the intensity or duration of iMBSR sessions may be beneficial. Future studies should consider the influence of nurses’ personality traits as a significant factor when evaluating the effects of psychological interventions on complex outcomes. Furthermore, it is advisable for managers and nurse policymakers to prioritize the physical health of nurses by addressing factors such as their interest in the nursing profession, satisfaction with the work environment, and mental health issues. This holistic approach can contribute to a more supportive and sustainable healthcare environment for nurses.

The findings also highlight the potential for telehealth-based interventions like iMBSR to be widely adopted in healthcare settings, particularly for populations with busy or unpredictable schedules, such as nurses. Telehealth interventions not only offer greater accessibility but also reduce the logistical challenges associated with in-person sessions. As the healthcare field continues to shift towards digital solutions, these results suggest that online platforms can be equally effective, if not more so, in delivering therapeutic interventions like MBSR. Future research should explore the broader implications of telehealth interventions for other clinical populations and assess whether the convenience and flexibility they offer consistently translate into improved outcomes across different contexts.

### 4.4 Limitations

The present study had several limitations that should be acknowledged. First, the small sample size in both intervention groups may limit the generalizability of the findings. Additionally, the absence of a control group made it difficult to definitively assess the effectiveness of MBSR and iMBSR in comparison to no intervention, which weakens the study’s ability to establish a causal relationship. The reliance on self-report measures also presents a potential limitation, as this can introduce bias or inaccuracies in reporting symptoms. Furthermore, the short follow-up period only allowed for the assessment of immediate effects, making it impossible to evaluate the long-term impact of MBSR and iMBSR on psychosomatic symptoms. A longer follow-up period would be necessary to determine whether the observed benefits are sustained over time. Future studies should address these limitations by including a control group, employing objective measures, and extending the follow-up duration to better assess long-term outcomes.

## 5 Conclusion

This study contributes to the growing body of evidence supporting both in-person and online MBSR interventions as effective tools for reducing depressive symptoms and improving mental health among nurses. Notably, the online MBSR intervention showed superior results in the GHQ scores compared to the face-to-face course, particularly in alleviating medically unexplained symptoms.

The reasons for the enhanced outcomes in the online format may be attributed to several underlying mechanisms. First, the flexibility and convenience of online courses can increase accessibility, allowing nurses to participate consistently despite demanding schedules. This convenience may also foster greater adherence, as participants can engage in sessions in a comfortable and personalized environment. Additionally, the structured and self-paced nature of online modules may facilitate a focused and individualized learning experience, potentially enhancing mindfulness practice. These factors highlight the importance of developing an impact model that explains why online courses might be more advantageous for certain populations, especially healthcare professionals with variable schedules.

The findings of this study hold significant implications for nursing leaders and healthcare organizations aiming to implement effective interventions to reduce stress, improve job satisfaction, and foster mental resilience among nurses. By integrating both traditional and telehealth-based mindfulness interventions into routine practice, healthcare organizations can better support nurses’ mental health, ultimately leading to improved patient care and lower turnover rates. This study emphasizes the need to promote a culture of self-care among nursing staff, prioritizing mental health as a cornerstone for a sustainable workforce.

Future research should focus on understanding the factors influencing the success of different intervention formats. Parameters like motivation to attend sessions, participant satisfaction, and engagement levels are critical to intervention research and should be systematically assessed. Longitudinal studies are necessary to explore the sustained effects of online MBSR and its broader applicability across diverse healthcare settings. Investigating the scalability and feasibility of telehealth-based mindfulness interventions can further support their integration into standard healthcare practice. Addressing these areas will enable a deeper understanding of the long-term benefits and the unique advantages that digital platforms might offer in promoting mental health and well-being in the nursing profession and beyond.

## Data Availability

The raw data supporting the conclusions of this article will be made available by the authors, without undue reservation.
